# New conditions for Pascal distribution series to be in a certain class of analytic functions

**DOI:** 10.1016/j.heliyon.2024.e40365

**Published:** 2024-11-17

**Authors:** Basem Aref Frasin, Luminiţa-Ioana Cotîrlă

**Affiliations:** a Department of Mathematics, Faculty of Science, Al al-Bayt University, Mafraq, Jordan; b Department of Mathematics, Technical University of Cluj-Napoca, 400114 Cluj-Napoca, Romania

**Keywords:** Analytic functions, Hadamard product, Stirling numbers, Pascal distribution series

## Abstract

Studies of Sălăgean differential operator $D^{\kappa }$ in connection with Stirling numbers are relatively new. In this paper, the differential operator $D^{\kappa }$ involving Stirling numbers is considered. New necessary and sufficient conditions involving Stirling numbers for the series ${ϒ}_{\theta }^{s}(\varsigma )$ written by the Pascal distribution are discussed for the subclass $T_{\kappa }(\epsilon ,\alpha )$. Also, we provide sufficient condition for the inclusion relation $I_{\theta }^{s}\left(R^{\varpi }(E,D\right))\subset T_{\kappa }(\epsilon ,\alpha )$. Further, we consider the properties of integral operator related to Pascal distribution series. New special cases as consequences of the main results are also obtained.

## 1 Introduction and definitions

Many new operators of analytic functions such as Alexander integral operator [1], the generalized Bernardi operator [2], Ruscheweyh derivative operator [3], Carlson-Shaffer operator [4], Sălăgean differential operator [5], Noor integral operator [6], Dziok-Srivastava operator [7], Al–Oboudi differential operator [8] and many others have been introduced and studied in the geometric function theory. Making use of these operators many researches introduced new subclasses of A of analytic functions defined in the disk Δ={ς∈C:|ς|<1} and written as

(1) $$f(\varsigma )=\varsigma +\sum_{\tau =2}^{\infty }a_{\tau }\varsigma^{\tau },$$

For a function f(ς) in A, we define

$$D^{0}f(\varsigma )=f(\varsigma ),$$

$$D^{1}f(\varsigma )=\varsigma f^{\prime }(\varsigma ),$$

and in general, we have

$$D^{\kappa }f(\varsigma )=\varsigma {\left(D^{\kappa -1}f(\varsigma )\right)}^{\prime },\;(\kappa \in N).$$

The differential operator $D^{\kappa }$ was introduced by Sălăgean [5].

Stirling number of the second kind [11] is the number of ways to partition a set of *κ* objects into *j* non-empty subsets and is denoted by $S_{\kappa ,j}$. Very recently, Frasin [9] (see also, [10]) noticed that the operator $D^{\kappa }$ can be written in terms of Stirling numbers as follows:

$$D^{\kappa }f(\varsigma )=\varsigma {\left(D^{\kappa -1}f(\varsigma )\right)}^{\prime },=S_{\kappa ,1}\varsigma f^{\prime }(\varsigma )+S_{\kappa ,2}\varsigma^{2}f^{\prime \prime }(\varsigma )+S_{\kappa ,3}\varsigma^{3}f^{\prime \prime \prime }(\varsigma )+\cdots +S_{\kappa ,\kappa }\varsigma^{\kappa }f^{\left(\kappa \right)}(\varsigma ),=\sum_{j=1}^{\kappa }S_{\kappa ,j}\varsigma^{j}f^{\left(j\right)}(\varsigma )\;(\kappa \in N),$$

where

$$S_{\kappa ,j}=jS_{\kappa -1,j}+S_{\kappa -1,j-1},\text{ and }S_{\kappa ,1}=S_{\kappa ,\kappa }=1.$$

For example,

(*i*) If κ=2, we have

$$D^{2}f(\varsigma )=\varsigma {\left(D^{1}f(\varsigma )\right)}^{\prime },=\varsigma f^{\prime }(\varsigma )+\varsigma^{2}f^{\prime \prime }(\varsigma ),=S_{2,1}\varsigma f^{\prime }(\varsigma )+S_{2,2}\varsigma^{2}f^{\prime \prime }(\varsigma ),$$

where

$$S_{2,1}=S_{2,2}=1.$$

(ii) If κ=3, we have

$$D^{3}f(\varsigma )=\varsigma {\left(D^{2}f(\varsigma )\right)}^{\prime },=\varsigma f^{\prime }(\varsigma )+3\varsigma^{2}f^{\prime \prime }(\varsigma )+\varsigma^{3}f^{\prime \prime \prime }(\varsigma ),=S_{3,1}\varsigma f^{\prime }(\varsigma )+S_{3,2}\varsigma^{2}f^{\prime \prime }(\varsigma )+S_{3,3}\varsigma^{3}f^{\prime \prime \prime }(\varsigma ),$$

where

$$S_{3,2}=2S_{2,2}+S_{2,1}=3,S_{3,1}=S_{3,3}=1.$$

(iii) If κ=4, we have

$$D^{4}f(\varsigma )=\varsigma {\left(D^{3}f(\varsigma )\right)}^{\prime },=\varsigma f^{\prime }(\varsigma )+7\varsigma^{2}f^{\prime \prime }(\varsigma )+6\varsigma^{3}f^{\prime \prime \prime }(\varsigma )+\varsigma^{4}f^{\left(4\right)}(\varsigma ),=S_{4,1}\varsigma f^{\prime }(\varsigma )+S_{4,2}\varsigma^{2}f^{\prime \prime }(\varsigma )+S_{4,3}\varsigma^{3}f^{\prime \prime \prime }(\varsigma )+S_{4,4}\varsigma^{4}f^{\left(4\right)}(\varsigma ),$$

where

$$S_{4,2}=2S_{3,2}+S_{3,1}=7,S_{4,3}=3S_{3,3}+S_{3,2}=6,S_{4,1}=S_{4,4}=1.$$

(iii) If κ=5, we have

$$D^{5}f(\varsigma )=\varsigma {\left(D^{4}f(\varsigma )\right)}^{\prime },=\varsigma f^{\prime }(\varsigma )+15\varsigma^{2}f^{\prime \prime }(\varsigma )+25\varsigma^{3}f^{\prime \prime \prime }(\varsigma )+10\varsigma^{4}f^{\left(4\right)}(\varsigma )+\varsigma^{5}f^{\left(5\right)}(\varsigma )=S_{5,1}\varsigma f^{\prime }(\varsigma )+S_{5,2}\varsigma^{2}f^{\prime \prime }(\varsigma )+S_{5,3}\varsigma^{3}f^{\prime \prime \prime }(\varsigma )+S_{5,4}\varsigma^{4}f^{\left(4\right)}(\varsigma )+S_{5,5}\varsigma^{5}f^{\left(5\right)}(\varsigma ),$$

where

$$S_{5,2}=2S_{4,2}+S_{4,1}=15,S_{5,3}=3S_{4,3}+S_{4,2}=25,S_{5,4}=4S_{4,4}+S_{4,3}=10,S_{5,1}=S_{5,5}=1.$$

Table 1 represents the coefficients $S_{\kappa ,j}$ of $\varsigma^{\kappa }\hbar^{\left(\kappa \right)}(\varsigma );1\leq \kappa \leq 6$.

**Table 1:** First few possibilities for Stirling numbers of the second kind (see [12]).

	*ςħ*′(*ς*)	*ς*^2^*ħ*″(*ς*)	*ς*^3^*ħ*^‴^(*ς*)	*ς*^4^*ħ*^(4)^(*ς*)	*ς*^5^*ħ*^(5)^(*ς*)	*ς*^6^*ħ*^(6)^(*ς*)
$D^{1}\hbar (\varsigma )$	1	0	0	0	0	0
$D^{2}\hbar (\varsigma )$	1	1	0	0	0	0
$D^{3}\hbar (\varsigma )$	1	3	1	0	0	0
$D^{4}\hbar (\varsigma )$	1	7	6	1	0	0
$D^{5}\hbar (\varsigma )$	1	15	25	10	1	0
$D^{6}\hbar (\varsigma )$	1	31	90	65	15	1
⋮	1	⋮	⋮	⋮	⋮	⋮
$D^{\kappa }\hbar (\varsigma )$	1	*S*_*κ*,2_	*S*_*κ*,3_	*S*_*κ*,4_	*S*_*κ*,5_	*S*_*κ*,6_

From this table we note that

1. $S_{\kappa ,j}=jS_{\kappa -1,j}+S_{\kappa -1,j-1}$
2. $S_{\kappa ,\kappa -1}=\frac{\kappa (\kappa -1)}{2}=\left(\begin{array}{c} \kappa \\ 2 \end{array}\right)$
3. $S_{\kappa ,2}=2^{\kappa -1}-1$
4. $S_{\kappa ,3}=\frac{1}{6}\left(3^{\kappa }-3.2^{\kappa }+3\right)$
5. $S_{\kappa ,1}=S_{\kappa ,\kappa }=1$
6. $S_{\kappa ,j}=0$, when j>κ
7. $S_{p,j\;}\equiv 0\;(\mathrm{mod}\;p)$ if 1<j<p; where *p* is a prime number.

Furthermore, for κ=2,3,4,5, we observe that

(2) $$D^{2}f(\varsigma )=\varsigma^{2}f^{\prime \prime }(\varsigma )+\varsigma f^{\prime }(\varsigma ),\varsigma +\sum_{\tau =2}^{\infty }\tau^{2}a_{\tau }\varsigma^{\tau }=\varsigma +\sum_{\tau =2}^{\infty }[\tau (\tau -1)+\tau ]a_{\tau }\varsigma^{\tau },$$

(3) $$D^{3}f(\varsigma )=\varsigma^{3}f^{\prime \prime \prime }(\varsigma )+3\varsigma^{2}f^{\prime \prime }(\varsigma )+\varsigma f^{\prime }(\varsigma ),\varsigma +\sum_{\tau =2}^{\infty }\tau^{3}a_{\tau }\varsigma^{\tau }=\varsigma +\sum_{\tau =2}^{\infty }[\tau (\tau -1)(\tau -2)+3\tau (\tau -1)+\tau ]a_{\tau }\varsigma^{\tau },$$

(4) $$D^{4}f(\varsigma )=\varsigma^{4}f^{\left(4\right)}(\varsigma )+6\varsigma^{3}f^{\prime \prime \prime }(\varsigma )+7\varsigma^{2}f^{\prime \prime }(\varsigma )+\varsigma f^{\prime }(\varsigma ),\varsigma +\sum_{\tau =2}^{\infty }\tau^{4}a_{\tau }\varsigma^{\tau }=\varsigma +\sum_{\tau =2}^{\infty }\left[\tau (\tau -1)(\tau -2)(\tau -3)+6\tau (\tau -1)(\tau -2\right)+7\tau (\tau -1)+\tau ],$$

and

(5) $$D^{5}f(\varsigma )=\varsigma^{5}f^{\left(5\right)}(\varsigma )+15\varsigma^{4}f^{\left(4\right)}(\varsigma )+25\varsigma^{3}f^{\prime \prime \prime }(\varsigma )+10\varsigma^{2}f^{\prime \prime }(\varsigma )+\varsigma f^{\prime }(\varsigma ),$$

From (2)-(5), we conclude that

$$\tau =(\tau -1)+1=S_{2,2}(\tau -1)+S_{2,1},\tau^{2}=(\tau -1)(\tau -2)+3(\tau -1)+1=S_{3,3}(\tau -1)(\tau -2)+S_{3,2}(\tau -1)+S_{3,1},\tau^{3}=(\tau -1)(\tau -2)(\tau -3)+6(\tau -1)(\tau -2)+7(\tau -1)+1=S_{4,4}(\tau -1)(\tau -2)(\tau -3)+S_{4,3}(\tau -1)(\tau -2)+S_{4,2}(\tau -1)+S_{4,1},$$

and

$$\tau^{4}=(\tau -1)(\tau -2)(\tau -3)(\tau -4)+10(\tau -1)(\tau -2)(\tau -3)+25(\tau -1)(\tau -2)+15(\tau -1)+1=S_{5,5}(\tau -1)(\tau -2)(\tau -3)(\tau -4)+S_{5,4}(\tau -1)(\tau -2)(\tau -3)+S_{5,3}(\tau -1)(\tau -2)+S_{5,2}(\tau -1)+S_{5,1}.$$

In general, we have

(6) $$\tau^{\kappa }=S_{\kappa +1,1}+S_{\kappa +1,2}(\tau -1)+S_{\kappa +1,3}(\tau -1)(\tau -2)+\cdots +S_{\kappa +1,\kappa +1}(\tau -1)(\tau -2)(\tau -3)\cdots (\tau -\kappa )=S_{\kappa +1,1}+\sum_{j=2}^{\kappa +1}S_{\kappa +1,j}(\tau -1)(\tau -2)(\tau -3)\cdots (\tau -(j-1)),\;\;\kappa =1,2,3,\cdots .$$

For functions f∈A given by (1) and g∈A given by $g(\varsigma )=\varsigma +\sum_{\tau =2}^{\infty }b_{\tau }\varsigma^{\tau }$, we recall that the well-known Hadamard (or convolution) product of *f* and *g* is given by

$$(f⁎g)(\varsigma ):=\varsigma +\sum_{\tau =2}^{\infty }a_{\tau }b_{\tau }\varsigma^{\tau },\;\varsigma \in \Delta .$$

In [13], Dixit and Pal introduced the following subclass of analytic functions

$$R^{\varpi }(E,D)=\{f\in A:\left|\frac{f^{\prime }(\varsigma )-1}{\left(E-D)\varpi -D[f^{\prime }(\varsigma )-1\right]}\right|<1,\;\;\;\;\varsigma \in \Delta ,$$

where ϖ∈C﹨{0} and −1≤D<E≤1.

Using the differential operator $D^{\kappa }$, we say that a function f(ς) belonging to A is said to be in the class $S_{\kappa }(\epsilon ,\alpha )$ if and only if

$$R\left\{\frac{\frac{D^{\kappa +1}f(\varsigma )}{D^{\kappa }f(\varsigma )}}{\epsilon \frac{D^{\kappa +1}f(\varsigma )}{D^{\kappa }f(\varsigma )}+(1-\epsilon )}\right\}>\alpha ,\;(\kappa \in N_{0}=N\cup \{0\}),$$

for some α(0≤α<1), ϵ(0≤ϵ<1) and for all *ς* in Δ.

Further, we define the class $T_{\kappa }(\epsilon ,\alpha )$ by

$$T_{\kappa }(\epsilon ,\alpha )=S_{\kappa }(\epsilon ,\alpha )\cap T\text{,}$$

where the subclass T of A consisting of functions of the form,

(7) $$f(\varsigma )=\varsigma -\sum_{\tau =2}^{\infty }\left|a_{\tau }\right|\varsigma^{\tau },\;\varsigma \in \Delta \text{.}$$

The class $T_{\kappa }(\epsilon ,\alpha )$ was introduced and studied by Aouf and Cho [14].

Clearly, we have $T_{0}(\epsilon ,\alpha )$ =T(ϵ,α) and $T_{1}(\epsilon ,\alpha )$ = C(ϵ,α) (see, [15]); $T_{0}(0,\alpha )$ = T ${}^{⁎}(\alpha )$ and $T_{1}(0,\alpha )$ = C(α) (see, [16]) and $T_{\kappa }(0,\alpha )$ = T(κ,α) (see, [17]).

The distributions of random variables have generated a great deal of interest in recent years. Their probability density functions (PDFs), in a real variable *x* and a complex variable *z*, have played an important role in statistics and probability theory.

A variable *X* is said to be Pascal distribution if it takes the values 0,1,2,3,… with probabilities ${\left(1-\theta \right)}^{s}$, $\frac{\theta s{\left(1-\theta \right)}^{s}}{1!}$, $\frac{\theta^{2}s(s+1){\left(1-\theta \right)}^{s}}{2!}$, $\frac{\theta^{3}s(s+1)(s+2){\left(1-\theta \right)}^{s}}{3!}$, …, respectively, where *θ* and s are called the parameters, and thus

$$P(X=\upsilon )=\left(\begin{array}{c} \upsilon +s-1\\ s-1 \end{array}\right)\;\theta^{\upsilon }{\left(1-\theta \right)}^{s},\;\;\;\upsilon =0,1,2,3,\cdots .$$

Let $\Lambda_{\theta }^{s}(\varsigma )$ be the following power series whose coefficients are probabilities of Pascal distribution [18]

$$\Lambda_{\theta }^{s}(\varsigma )=\varsigma +\sum_{\tau =2}^{\infty }\left(\begin{array}{c} \tau +s-2\\ s-1 \end{array}\right)\theta^{\tau -1}{\left(1-\theta \right)}^{s}\varsigma^{\tau },\;\varsigma \in \Delta$$

where s≥1;0≤θ≤1 and we note that, by ratio test the radius of convergence of above series is infinity. We also define the series

(8) $${ϒ}_{\theta }^{s}(\varsigma )=2\varsigma -\Lambda_{\theta }^{s}(\varsigma )=\varsigma -\sum_{\tau =2}^{\infty }\left(\begin{array}{c} \tau +s-2\\ s-1 \end{array}\right)\theta^{\tau -1}{\left(1-\theta \right)}^{s}\varsigma^{\tau },\;\varsigma \in \Delta .$$

Now, we consider the linear operator (see, [18])

$$I_{\theta }^{s}f(\varsigma ):A\to A$$

defined by the convolution or Hadamard product

$${\;I}_{\theta }^{s}f(\varsigma )=\Lambda_{\theta }^{s}(\varsigma )⁎f(\varsigma )=\varsigma +\sum_{\tau =2}^{\infty }\left(\begin{array}{c} \tau +s-2\\ s-1 \end{array}\right)\theta^{\tau -1}{\left(1-\theta \right)}^{s}a_{\tau }\varsigma^{\tau },\;\varsigma \in \Delta .$$

In recent years, several researchers used Pascal distribution series and other distribution series such as Poisson distribution series, hypergeometric distribution series, and the Mittag–Leffler-type Poisson distribution to obtain some necessary and sufficient conditions for these distributions to belong to certain classes of analytic functions defined in the open unit disk (see for example, [19], [20], [21], [22], [23], [24], [25], [26], [27], [28], [29], [30], [31], [32], [33], [34], [35], [36], [37], [38], [39]).

The paper is organized as follows. In Section 2, we recall some lemmas and useful relations, which will be useful to prove the main results. Section 3 is devoted to obtain the necessary and sufficient condition for ${ϒ}_{\theta }^{s}(\varsigma )$ to be in the class $T_{\kappa }(\epsilon ,\alpha )$. In Section 4, we give sufficient condition for ${\;I}_{\theta }^{s}\left(R^{\varpi }(E,D\right))\subset T_{\kappa }(\epsilon ,\alpha )$. Finally, in Section 5, we give conditions for the integral operator $G_{\theta }^{s}f(\varsigma )=\int_{0}^{\varsigma }\frac{{ϒ}_{\theta }^{s}(t)}{t}dt$ to be in the class $T_{\kappa }(\epsilon ,\alpha )$.

## 2 Lemmas and useful relations

By simple calculations we derive the following relations:

$$\sum_{\tau =2}^{\infty }\left(\begin{array}{c} \tau +s-2\\ s-1 \end{array}\right)\;\theta^{\tau -1}=\frac{1}{{\left(1-\theta \right)}^{s}}-1,$$

$$\sum_{\tau =5}^{\infty }(\tau -1)(\tau -2)(\tau -3)(\tau -4)\left(\begin{array}{c} \tau +s-2\\ s-1 \end{array}\right)\;\theta^{\tau -1}=\frac{24\theta^{4}\left(\begin{array}{c} s+3\\ s-1 \end{array}\right)}{{\left(1-\theta \right)}^{s+4}},\sum_{\tau =4}^{\infty }(\tau -1)(\tau -2)(\tau -3)\left(\begin{array}{c} \tau +s-2\\ s-1 \end{array}\right)\;\theta^{\tau -1}=\frac{6\theta^{3}\left(\begin{array}{c} s+2\\ s-1 \end{array}\right)}{{\left(1-\theta \right)}^{s+3}},\sum_{\tau =3}^{\infty }(\tau -1)(\tau -2)\left(\begin{array}{c} \tau +s-2\\ s-1 \end{array}\right)\;\theta^{\tau -1}=\frac{2\theta^{2}\left(\begin{array}{c} s+1\\ s-1 \end{array}\right)}{{\left(1-\theta \right)}^{s+2}},\sum_{\tau =2}^{\infty }(\tau -1)\left(\begin{array}{c} \tau +s-2\\ s-1 \end{array}\right)\;\theta^{\tau -1}=\frac{\theta \left(\begin{array}{c} s\\ s-1 \end{array}\right)}{{\left(1-\theta \right)}^{s+1}},$$

and in general, for s=1,2,3,⋯, we have

(9) $$\sum_{\tau =s+1}^{\infty }(\tau -1)(\tau -2)(\tau -3)\cdots (\tau -s)\left(\begin{array}{c} \tau +s-2\\ s-1 \end{array}\right)\;\theta^{\tau -1}=s!\theta^{s}\frac{\left(\begin{array}{c} s+s-1\\ s-1 \end{array}\right)}{{\left(1-\theta \right)}^{s+s}}.$$

To prove our main results, we need the following lemmas.

Lemma 2.1 *[14]* *Let the function* f(ς) *be defined by* (7) *. Then* f(ς)∈ $T_{\kappa }(\epsilon ,\alpha )$ *if and only if*

(10) $$\sum_{\tau =2}^{\infty }\tau^{\kappa }\left(\tau -\alpha [1+\epsilon (\tau -1)]\right)\left|a_{\tau }\right|\leq 1-\alpha ,\;\;\;\;\;(\varsigma \in \Delta ).$$

*The result is sharp.*

Lemma 2.2 *[13]**If f* ∈ $R^{\varpi }(E,D)$ *is of the form* (1)*, then*

(11) $$\left|a_{\tau }\right|\leq (E-D)\frac{\left|\varpi \right|}{\tau },\;\;\;\;\;\;\varpi \in N-\{1\}\text{.}$$

*The result is sharp.*

Unless otherwise mentioned, we shall assume in this paper that 0≤α<1, 0≤ϵ<1 s≥1 and 0≤θ<1.

## 3 The necessary and sufficient conditions

First of all, with the help of Lemma 2.1, we obtain the following necessary and sufficient condition for ${ϒ}_{\theta }^{s}(\varsigma )$ to be in $T_{\kappa }(\epsilon ,\alpha )$.

Theorem 3.1 *The series* ${ϒ}_{\theta }^{s}(\varsigma )\in T_{\kappa }(\epsilon ,\alpha )$ *,* κ≥1 *, if and only if*

(12) $$(1-\alpha \epsilon )(\kappa +1)!\theta^{\kappa +1}\frac{\left(\begin{array}{c} s+\kappa \\ s-1 \end{array}\right)}{{\left(1-\theta \right)}^{s+\kappa +1}}+\sum_{j=2}^{\kappa +1}\left((1-\alpha \epsilon )S_{\kappa +2,j}-(\alpha -\alpha \epsilon )S_{\kappa +1,j}\right)\left((j-1)!\theta^{j-1}\frac{\left(\begin{array}{c} s+j-2\\ s-1 \end{array}\right)}{{\left(1-\theta \right)}^{s+j-1}}\right)\leq 1-\alpha .$$

Proof In view of (10), we will show that P≤1−α, where

$$P=\sum_{\tau =2}^{\infty }\tau^{\kappa }\left(\tau -\alpha [1+\epsilon (\tau -1)]\right)\left(\begin{array}{c} \tau +s-2\\ s-1 \end{array}\right)\theta^{\tau -1}{\left(1-\theta \right)}^{s}.$$

Using (6) and (9), we have

$$P=\sum_{\tau =2}^{\infty }[\tau^{\kappa +1}(1-\alpha \epsilon )-(\alpha -\alpha \epsilon )\tau^{\kappa }]\left(\begin{array}{c} \tau +s-2\\ s-1 \end{array}\right)\theta^{\tau -1}{\left(1-\theta \right)}^{s}=\sum_{\tau =2}^{\infty }[\left((1-\alpha \epsilon )+(1-\alpha \epsilon )S_{\kappa +2,\kappa +2}\;(\tau -1)(\tau -2)\cdots (\tau -(\kappa +1))\right)+(1-\alpha \epsilon )\sum_{j=2}^{\kappa +1}S_{\kappa +2,j}\;(\tau -1)(\tau -2)\cdots (\tau -(j-1))-(\alpha -\alpha \epsilon )\left(1+\sum_{j=2}^{\kappa +1}S_{\kappa +1,j}\;(\tau -1)(\tau -2)\cdots (\tau -(j-1))\right)]\left(\begin{array}{c} \tau +s-2\\ s-1 \end{array}\right)\theta^{\tau -1}{\left(1-\theta \right)}^{s}={\left(1-\theta \right)}^{s}[(1-\alpha \epsilon )\sum_{\tau =\kappa +2}^{\infty }\;(\tau -1)(\tau -2)(\tau -3)\cdots (\tau -(\kappa +1))\left(\begin{array}{c} \tau +s-2\\ s-1 \end{array}\right)\theta^{\tau -1}+\sum_{j=2}^{\kappa +1}\left((1-\alpha \epsilon )S_{\kappa +2,j}-(\alpha -\alpha \epsilon )S_{\kappa +1,j}\right)\sum_{\tau =j}^{\infty }(\tau -1)(\tau -2)\cdots (\tau -(j-1))\left(\begin{array}{c} \tau +s-2\\ s-1 \end{array}\right)\theta^{\tau -1}+(1-\alpha )\sum_{\tau =2}^{\infty }\left(\begin{array}{c} \tau +s-2\\ s-1 \end{array}\right)\theta^{\tau -1}]=(1-\alpha \epsilon )(\kappa +1)!\theta^{\kappa +1}\frac{\left(\begin{array}{c} s+\kappa \\ s-1 \end{array}\right)}{{\left(1-\theta \right)}^{\kappa +1}}+\sum_{j=2}^{\kappa +1}\left((1-\alpha \epsilon )S_{\kappa +2,j}-(\alpha -\alpha \epsilon )S_{\kappa +1,j}\right)\left((j-1)!\theta^{j-1}\frac{\left(\begin{array}{c} s+j-2\\ s-1 \end{array}\right)}{{\left(1-\theta \right)}^{j-1}}\right)+(1-\alpha )\left(1-{\left(1-\theta \right)}^{s}\right).$$

Therefore, we see that the last expression is bounded above by 1−α if (12) is satisfied. □

Putting κ=1 in Theorem 3.1, we obtain the following corollary.

Corollary 3.2 *The series*${ϒ}_{\theta }^{s}(\varsigma )$ ∈ C(ϵ,α)*, if and only if*

$$(1-\alpha \epsilon )\theta^{2}\frac{s(s+1)}{{\left(1-\theta \right)}^{s+2}}+\left(3-\alpha -2\alpha \epsilon )\right)\left(\theta \frac{s}{{\left(1-\theta \right)}^{s+1}}\right)\leq 1-\alpha .$$

Putting κ=2 in Theorem 3.1, we obtain the following corollary.

Corollary 3.3 *The series*${ϒ}_{\theta }^{s}(\varsigma )$ ∈ $T_{2}(\epsilon ,\alpha )$*, if and only if*

$$(1-\alpha \epsilon )\frac{\theta^{3}s(s+1)(s+2)}{{\left(1-\theta \right)}^{s+3}}+\left(6-5\alpha \epsilon -\alpha \right)\frac{\theta^{2}s(s+1)}{{\left(1-\theta \right)}^{s+2}}+\left(7-4\alpha \epsilon -3\alpha \right)\frac{\theta s}{{\left(1-\theta \right)}^{s+1}}\leq 1-\alpha .$$

Putting ϵ=0 in Corollary 3.2, we obtain the following corollary.

Corollary 3.4 *The series*${ϒ}_{\theta }^{s}(\varsigma )$ ∈ C(α)*, if and only if*

$$\theta^{2}\frac{s(s+1)}{{\left(1-\theta \right)}^{s+2}}+\left(3-\alpha \right)\theta \frac{s}{{\left(1-\theta \right)}^{s+1}}\leq 1-\alpha .$$

Putting ϵ=0 in Theorem 3.1, we obtain the following corollary.

Corollary 3.5 *The series*${ϒ}_{\theta }^{s}(\varsigma )$ ∈ T(κ,α)*,* κ≥1*, if and only if*

$$((\kappa +1)!\theta^{\kappa +1}\frac{\left(\begin{array}{c} s+\kappa \\ s-1 \end{array}\right)}{{\left(1-\theta \right)}^{s+\kappa +1}}+\sum_{j=2}^{\kappa +1}\left(S_{\kappa +2,j}-\alpha S_{\kappa +1,j}\right)\left((j-1)!\theta^{j-1}\frac{\left(\begin{array}{c} s+j-2\\ s-1 \end{array}\right)}{{\left(1-\theta \right)}^{s+j-1}}\right)\leq 1-\alpha .$$

## 4 Inclusion properties

Making use of Lemma 2.2, we will study the inclusion relation $R^{\varpi }(E,D)\subset$ $T_{\kappa }(\epsilon ,\alpha )$.

Theorem 4.1 *Let* $f\in R^{\varpi }(E,D)$ *. Then* $I_{\theta }^{s}f(\varsigma )\in T_{\kappa }(\epsilon ,\alpha )$ *,* κ≥2 *, if*

$$(E-D)\left|\varpi \right|[(1-\alpha \epsilon )\kappa !\theta^{\kappa }\frac{\left(\begin{array}{c} s+\kappa -1\\ s-1 \end{array}\right)}{{\left(1-\theta \right)}^{\kappa }}$$

$$+\sum_{j=2}^{\kappa }\left((1-\alpha \epsilon )\;S_{\kappa +1,j}-(\alpha -\alpha \epsilon )S_{\kappa ,j}\right)\left((j-1)!\theta^{j-1}\frac{\left(\begin{array}{c} s+j-2\\ s-1 \end{array}\right)}{{\left(1-\theta \right)}^{j-1}}\right)+(1-\alpha )\left(1-{\left(1-\theta \right)}^{s}\right)]$$

(13) ≤1−α.

Proof From (10), it suffices to show that L≤1−α, where

(14) $$L=\sum_{\tau =2}^{\infty }\tau^{\kappa }\left(\tau -\alpha [1+\epsilon (\tau -1)]\right)\left(\begin{array}{c} \tau +s-2\\ s-1 \end{array}\right)\theta^{\tau -1}{\left(1-\theta \right)}^{s}\left|a_{\tau }\right|.$$

Applying (11) of Lemma 2.2, we find from equations (6) and (9) that

$$L\leq (E-D)\left|\varpi \right|\left[\sum_{\tau =2}^{\infty }\left((1-\alpha \epsilon )\tau^{\kappa }-(\alpha -\alpha \epsilon )\tau^{\kappa -1}\right)\left(\begin{array}{c} \tau +s-2\\ s-1 \end{array}\right)\theta^{\tau -1}{\left(1-\theta \right)}^{s}\right]\leq (E-D)\left|\varpi \right|{\left(1-\theta \right)}^{s}[\sum_{\tau =2}^{\infty }\left[(1-\alpha \epsilon )+(1-\alpha \epsilon )S_{\kappa +1,\kappa +1}(\tau -1)(\tau -2)\cdots (\tau -\kappa \right)+(1-\alpha \epsilon )\sum_{j=2}^{\kappa }S_{\kappa +1,j}\;(\tau -1)(\tau -2)\cdots (\tau -(j-1))-(\alpha -\alpha \epsilon )\left(1+\sum_{j=2}^{\kappa }S_{\kappa ,j}(\tau -1)(\tau -2)\cdots (\tau -(j-1))\right)]]\left(\begin{array}{c} \tau +s-2\\ s-1 \end{array}\right)\theta^{\tau -1}\leq (E-D)\left|\varpi \right|{\left(1-\theta \right)}^{s}[(1-\alpha \epsilon )\sum_{\tau =\kappa +1}^{\infty }(\tau -1)(\tau -2)\cdots (\tau -\kappa )\left(\begin{array}{c} \tau +s-2\\ s-1 \end{array}\right)\theta^{\tau -1}+\sum_{j=2}^{\kappa }\left((1-\alpha \epsilon )S_{\kappa +1,j}\;-(\alpha -\alpha \epsilon )S_{\kappa ,j}\right)\sum_{\tau =j}^{\infty }(\tau -1)(\tau -2)\cdots (\tau -(j-1))\left(\begin{array}{c} \tau +s-2\\ s-1 \end{array}\right)\theta^{\tau -1}+(1-\alpha )\sum_{\tau =2}^{\infty }\left(\begin{array}{c} \tau +s-2\\ s-1 \end{array}\right)\theta^{\tau -1}]\leq (E-D)\left|\varpi \right|[(1-\alpha \epsilon )\kappa !\theta^{\kappa }\frac{\left(\begin{array}{c} s+\kappa -1\\ s-1 \end{array}\right)}{{\left(1-\theta \right)}^{\kappa }}+\sum_{j=2}^{\kappa }\left((1-\alpha \epsilon )S_{\kappa +1,j}\;-(\alpha -\alpha \epsilon )S_{\kappa ,j}\right)\left((j-1)!\theta^{j-1}\frac{\left(\begin{array}{c} s+j-2\\ s-1 \end{array}\right)}{{\left(1-\theta \right)}^{j-1}}\right)+(1-\alpha )\left(1-{\left(1-\theta \right)}^{s}\right)].$$

But this last expression is bounded by 1−α, if (13) holds. □

Putting κ=2 in Theorem 4.1, we obtain the following corollary.

Corollary 4.2 *Let* $f\in R^{\varpi }(E,D)$ *. Then* $I_{\theta }^{s}f(\varsigma )\in T_{2}(\epsilon ,\alpha )$ *, if*

$$(E-D)\left|\varpi \right|[(1-\alpha \epsilon )\theta^{2}\frac{s(s+1)}{{\left(1-\theta \right)}^{2}}+(3-2\alpha \epsilon -\alpha )\theta \frac{s}{\left(1-\theta \right)}+(1-\alpha )\left(1-{\left(1-\theta \right)}^{s}\right)]\leq 1-\alpha .$$

Putting ϵ=0 in Theorem 4.1, we obtain the following corollary.

Corollary 4.3 *Let* $f\in R^{\varpi }(E,D)$ *. Then* $I_{\theta }^{s}f(\varsigma )\in T(\kappa ,\alpha )$ *,* κ≥2 *, if*

$$(E-D)\left|\varpi \right|[\kappa !\theta^{\kappa }\frac{\left(\begin{array}{c} s+\kappa -1\\ s-1 \end{array}\right)}{{\left(1-\theta \right)}^{\kappa }}+\sum_{j=2}^{\kappa }\left(\;S_{\kappa +1,j}-\alpha S_{\kappa ,j}\right)\left((j-1)!\theta^{j-1}\frac{\left(\begin{array}{c} s+j-2\\ s-1 \end{array}\right)}{{\left(1-\theta \right)}^{j-1}}\right)+(1-\alpha )\left(1-{\left(1-\theta \right)}^{s}\right)].\leq 1-\alpha .$$

## 5 An integral operator

In this section, we consider the integral operator $G_{\theta }^{s}$ defined by

(15) $$G_{\theta }^{s}f(\varsigma )=\int_{0}^{\varsigma }\frac{{ϒ}_{\theta }^{s}(t)}{t}dt.$$

Theorem 5.1 *The integral operator* $G_{\theta }^{s}f(\varsigma )$ *defined by* (15) *is in the class* $T_{\kappa }(\epsilon ,\alpha )$ *,* κ≥2 *, if and only if*

(16) $$(1-\alpha \epsilon )\kappa !\theta^{\kappa }\frac{\left(\begin{array}{c} s+\kappa -1\\ s-1 \end{array}\right)}{{\left(1-\theta \right)}^{\kappa }}+\sum_{j=2}^{\kappa }\left((1-\alpha \epsilon )S_{\kappa +1,j}\;-(\alpha -\alpha \epsilon )S_{\kappa ,j}\right)\left((j-1)!\theta^{j-1}\frac{\left(\begin{array}{c} s+j-2\\ s-1 \end{array}\right)}{{\left(1-\theta \right)}^{j-1}}\right)+(1-\alpha )\left(1-{\left(1-\theta \right)}^{s}\right)\leq 1-\alpha .$$

Proof From the definitions (8) and (15), it easily verified that

$$G_{\theta }^{s}f(\varsigma )=\varsigma -\sum_{\tau =2}^{\infty }\left(\begin{array}{c} \tau +s-2\\ s-1 \end{array}\right)\;\theta^{\tau -1}{\left(1-\theta \right)}^{s}\frac{\varsigma^{\tau }}{\tau }$$

then by Lemma 2.1, we need only to show that H≤1−α, where

$$H=\sum_{\tau =2}^{\infty }\tau^{\kappa }\left(\tau -\alpha [1+\epsilon (\tau -1)]\right)\times \frac{1}{\tau }\left(\begin{array}{c} \tau +s-2\\ s-1 \end{array}\right)\;\theta^{\tau -1}{\left(1-\theta \right)}^{s},$$

or equivalently,

$$H=\sum_{\tau =2}^{\infty }\left((1-\alpha \epsilon )\tau^{\kappa }-(\alpha -\alpha \epsilon )\tau^{\kappa -1}\right)\left(\begin{array}{c} \tau +s-2\\ s-1 \end{array}\right)\theta^{\tau -1}{\left(1-\theta \right)}^{s}.$$

We omit the details of the remaining part of the proof of Theorem 5.1 because it is similar to that of Theorem 4.1. □

Putting κ=2 in Theorem 5.1, we obtain the following corollary.

Corollary 5.2 *The integral operator* $G_{\theta }^{s}f(\varsigma )$ *defined by* (15) *is in the class* $T_{2}(\epsilon ,\alpha )$ *if and only if*

$$\frac{\left(1-\alpha \epsilon )\theta^{2}s(s+1\right)}{{\left(1-\theta \right)}^{s+2}}+(3-2\alpha \epsilon -\alpha )\frac{\theta s}{{\left(1-\theta \right)}^{s+1}}\leq 1-\alpha .$$

Putting ϵ=0 in Theorem 5.1, we obtain the following corollary.

Corollary 5.3 *The integral operator* $G_{\theta }^{s}f(\varsigma )$ *defined by* (15) *is in the class* T(κ,α) *,* κ≥2 *, if and only if*

$$\kappa !\theta^{\kappa }\frac{\left(\begin{array}{c} s+\kappa -1\\ s-1 \end{array}\right)}{{\left(1-\theta \right)}^{\kappa }}+\sum_{j=2}^{\kappa }\left(S_{\kappa +1,j}\;-\alpha S_{\kappa ,j}\right)\left((j-1)!\theta^{j-1}\frac{\left(\begin{array}{c} s+j-2\\ s-1 \end{array}\right)}{{\left(1-\theta \right)}^{j-1}}\right)+(1-\alpha )\left(1-{\left(1-\theta \right)}^{s}\right)\leq 1-\alpha .$$

Remark 5.4 Taking different choices of κ∈N, one can obtain new necessary and sufficient conditions and inclusion relations for Pascal distribution series to be in the class $T_{\kappa }(\epsilon ,\alpha )$.

## 6 Conclusions

In the literature there are many classes of analytic functions with negative coefficients have defined by the Sălăgean differential operator $D^{\kappa }$. In the present paper, we obtained new conditions for Pascal distribution series to be in the class $T_{\kappa }(\epsilon ,\alpha )$ of analytic functions defined by the operator $D^{\kappa }$ including Stirling numbers. In addition, we proved new conditions for the operator $G_{\theta }^{s}$ to be in the class $T_{\kappa }(\epsilon ,\alpha )$. Some special cases of our main results are also obtained. This study could inspire researchers to find new conditions and inclusion relations for Pascal distribution series to be in other classes defined by the operator $D^{\kappa }$.

## Ethical approval

Not applicable.

## CRediT authorship contribution statement

**Basem Aref Frasin:** Writing – review & editing, Writing – original draft, Visualization, Validation, Resources, Methodology, Investigation, Formal analysis, Data curation, Conceptualization. **Luminiţa-Ioana Cotîrlă:** Writing – review & editing, Writing – original draft, Visualization, Validation, Supervision, Software, Resources, Project administration, Methodology, Investigation, Funding acquisition, Formal analysis, Data curation, Conceptualization.

## Declaration of Competing Interest

The authors declare that they have no known competing financial interests or personal relationships that could have appeared to influence the work reported in this paper.

## Data Availability

Data included in article/supp. material/referenced in article.
